# Incidence, Etiology and Risk Factors for Travelers’ Diarrhea during a Hospital Ship-Based Military Humanitarian Mission: Continuing Promise 2011

**DOI:** 10.1371/journal.pone.0154830

**Published:** 2016-05-12

**Authors:** Jessica M. Hameed, Ramona L. McCaffrey, Andrea McCoy, Tracy Brannock, Gregory J. Martin, William T. Scouten, Krista Brooks, Shannon D. Putnam, Mark S. Riddle

**Affiliations:** 1 Department of Preventive Medicine & Biostatistics, Uniformed Services University of the Health Sciences, Bethesda, MD, United States of America; 2 Enteric Disease Surveillance Program, Operational Infectious Disease Department, Naval Health Research Center, San Diego, CA, United States of America; 3 Enteric Diseases Department, Infectious Disease Directorate, Naval Medical Research Center, Silver Spring, MD, United States of America; 4 Air Force Global Strike Command, Barksdale Air Force Base, LA, United States of America; 5 Department of State, Washington, DC, United States of America; 6 Naval Medical Center Portsmouth, Portsmouth, VA, United States of America; 7 Yayasan – International Health Development Foundation, Bali, Indonesia; University of Hyderabad, INDIA

## Abstract

Travelers’ diarrhea (TD) is the most common ailment affecting travelers, including deployed U.S. military. Continuing Promise 2011 was a 5-month humanitarian assistance/disaster response (HA/DR) military and non-governmental organization training mission aboard the hospital ship USNS Comfort, which deployed to Central and South America and the Caribbean between April and September 2011. Enhanced TD surveillance was undertaken during this mission for public health purposes. Passive surveillance (clinic visits), active surveillance (self-reported questionnaires), and stool samples were collected weekly from shipboard personnel. Descriptive statistics and multivariate-logistic regression methods were used to estimate disease burden and risk factor identification. Two polymerase chain reaction methods on frozen stool were used for microbiological identification. TD was the primary complaint for all clinic visits (20%) and the leading cause of lost duties days due to bed rest confinement (62%), though underreported, as the active self-reported incidence was 3.5 times higher than the passive clinic-reported incidence. Vomiting (p = 0.002), feeling lightheaded or weak (p = 0.005), and being a food handler (p = 0.017) were associated with increased odds of lost duty days. Thirty-eight percent of self-reported cases reported some amount of performance impact. Based on the epidemiological curve, country of exercise and liberty appeared to be temporally associated with increased risk. From the weekly self-reported questionnaire risk factor analysis, eating off ship in the prior week was strongly associated (adjusted odds ratio [OR] 2.4, p<0.001). Consumption of seafood increased risk (aOR 1.7, p = 0.03), though consumption of ice appeared protective (aOR 0.3, p = 0.01). Etiology was bacterial (48%), with enterotoxigenic *Escherichia coli* as the predominant pathogen (35%). Norovirus was identified as a sole pathogen in 12%, though found as a copathogen in an additional 6%. Despite employment of current and targeted preventive interventions, ship-board HA/DR missions may experience a significant risk for TD among deployed US military personnel and potentially impact mission success.

## Introduction

Travelers’ diarrhea (TD) is a frequent ailment affecting both military and civilian travelers to regions around the world where sanitation and hygiene standards are poor. The U.S. Centers for Disease Control and Prevention estimates that approximately 10 million people develop TD every year [1], and U.S. military estimates report an attack rate of 29 TD cases per every 100 person-months.[2] The most significant risk factor for TD is travel destination and type of travel, with Asia, the Middle East, Africa, Mexico, and Central and South America being the highest-risk regions, and backpackers, trekkers and visitors of friends and relatives being the highest at-risk populations.[3] From the sailing vessel and military perspectives, risk is associated with dwelling within densely populated living conditions and exposure to contaminated food and water sources.[4–14]

The hospital ship and humanitarian assistance/disaster response (HA/DR) missions represent a unique deployment environment in terms of exposures (intensive interaction with local populations) and occupation (health care providers). Understanding the burden of acute diarrheal disease among a hospital ship deployment may be even more of a challenge given that many medical personnel are not likely to report for care through normal clinical care channels and instead would tend to resort to self-care or care from colleagues. As such, this is the first study aimed to describe the incidence, impact, and etiology of acute diarrhea and gastroenteritis affecting the crew aboard the USNS Comfort (T-AH 20) during the 5-month Continuing Promise 2011 HA/DR mission to Central and South America. [15]

## Materials and Methods

### Study design

This study assessed data collected from multiple sources, including: (1) Disease and Non-battle Injury (DNBI) weekly aggregate reports of patients seeking care at the USNS Comfort’s medical clinic for symptoms/syndromes consistent with acute infectious gastroenteritis; (2) a weekly, serial, cross-sectional sampling of the ship’s population via an anonymous and voluntary questionnaire; and (3) standardized clinical case series data from patients with acute diarrhea or gastroenteritis of presumed infectious etiology that included a subset of cases tested via culture-independent methods to describe specific infectious etiology. These data were collected for the primary purpose of public health surveillance. Notably, all data collected were de-identified (i.e., no social security numbers, names, addresses, telephone numbers, or dates of birth were provided at any time), and no subjects were later contacted as part of the study. For reporting purposes, this analysis project was approved by the Uniformed Services University Office of Research as exempt.

### Study population

The study population included the entire USNS Comfort ship’s crew, including civil service Merchant Marines from the Military Sealift Command, active duty military (primarily U.S. Navy), and civilian volunteers associated with multiple non-governmental organizations (NGOs). The ships population changed weekly due to rotating personnel embarking and disembarking in support of the mission at each of the country stops. Based on personnel data the weekly average was 910 persons (standard deviation 15, range 884–937).

### Epidemiologic methods

Weekly de-identified clinical aggregate data were compiled as mandated by the Department of Defense for Force Health Protection and public health surveillance for total and categorical DNBI including time aboard ship.[16] In addition to this passive case identification via the ship’s medical clinic, cases were also identified actively via anonymous, self-reported questionnaires that were completed by ~15% of the ship’s population. In this active surveillance effort, a questionnaire (S1 File) was completed on a weekly basis among those who were aboard ship. The questionnaire was distributed on the same day each week (Friday) and sampled personnel going off-ship as they staged each morning for departure, as well as 15% of the medical, nursing, administrative, and surgical departments’ personnel who normally would stay on ship. On days where there were no missions involving personnel going off ship (e.g. ship was underway), 15% of the ships population proportional to the size of the main departments were provided forms to complete during their routine morning muster. The form was designed to be completed quickly and capture demographic information, country visited, off-ship exposure, illness and injury events, medical care sought, food/drink consumed off-ship, and illness impact on the individual’s ability to perform the mission. These forms were collected and entered into a database for weekly trend analysis and reporting to the Medical Department and Command to use as information to support Force Health Protection.

In addition to these epidemiological methods, a case series was performed on all personnel presenting to the ship’s clinic for travelers’ diarrhea. The term travelers’ diarrhea was used to encompass both acute diarrhea and gastroenteritis cases of presumed infectious etiology and defined as: (1) three or more episodes of loose or liquid stools within a 24-hour period; (2) two or more episodes of liquid stools accompanied by either nausea, vomiting, abdominal cramping, fever, chills, muscle aches, joint pains, or urgency; or (3) two or more episodes of vomiting in the past 24 hours that could not be explained by non-infectious etiologies [17]. A standardized case report form was completed for cases seen within the ship’s medical clinic that included specific GI and systemic infection symptomatology fields as well as collection of standard medical metrics, including vital signs, exam procedures, and treatments.

### Culture-independent microbiological testing

Stool samples were collected from a subset of cases (~ 5 initial cases per country) that were treated in the ship’s medical clinic and met the above case definition for infectious diarrhea/gastroenteritis. Subset sampling was performed because both personnel time and personnel and material resources were limited. After a stool sample was collected, it was stored at -70°C aboard ship. Samples were then shipped on dry ice to the Naval Health Research Center (San Diego, CA) Enteric Disease Surveillance Program laboratory. Conventional, real-time, and Luminex xTAG^®^-Gastrointestinal Pathogen Panel (GPP)-based (reverse transcription) polymerase chain reaction (RT-)PCR was performed to detect various bacterial, viral, and parasitic enteric pathogens within clinical samples (Luminex Corporation, Toronto, Canada).

Briefly, frozen stool samples were diluted to a 20% solution with phosphate-buffered saline and subjected to nucleic acid extraction using the Qiagen QIAamp Viral RNA Mini Kit or the QIAamp Fast DNA Stool Mini Kit (Qiagen, Valencia, CA). Sample RNAs/DNAs were then subjected to conventional and real-time (RT-)PCR for identification. The conventional PCR assayed for diarrheagenic *Escherichia coli* (including enterotoxigenic *E*. *coli* [ETEC], enteroinvasive *E*. *coli* [EIEC], enteropathogenic *E*. *coli* [EPEC] and enteroaggregative *E*. *coli* [EAEC]), *Salmonella* species (spp.), *Shigella* spp., *Campylobacter* spp., *Vibrio cholerae*, astrovirus, groups A and C rotavirus, sapovirus, adenovirus, and norovirus genogroups GI and GII (S2 File). Identification of norovirus was performed using a real-time RT-PCR protocol stipulated by the CDC CaliciNet program. [18] The commercial FDA approved multiplex assay, Luminex xTAG^®^ GPP-based pathogen identification assay was also performed per manufacturer’s instructions and included tests for the following pathogens: *Campylobacter* spp. *(C*. *jejuni*, *C*. *coli*, *C*. *lari*, *Clostridium difficile* toxin A/B, Shiga-like toxin producing *E*. *coli* (STEC), *E*. *coli* O157, ETEC, *Salmonella* spp., *Shigella* spp. *(S*. *boydii*, *S*. *dysenteriae*, *S*. *flexneri*, *S*. *sonnei*, NoV (GI/GII), rotavirus A, *Cryptosporidium parvum*, *C*. *hominis*, *Entamoeba histolytica*, and *Giardia lamblia*.

### Statistical analysis

Weekly TD incidence rate was calculated for both clinic-associated cases and TD cases reported by the population sampled from the weekly anonymous survey. The numerator for each of these calculations was the sum of the number of TD case events divided by the weekly person-time (e.g. weekly ship-board population for clinic-associated cases and total surveys completed for self-reported DNBI reporting) to estimate the incidence rate along with 95% confidence interval (CI) obtained using a Mid-P exact test via the *Open Epi* online statistical program.[19] Descriptive statistics, mean difference, Student’s t-test, Fisher’s exact tests, chi-square tests, and multivariate logistic regression tests were calculated using SPSS Statistics for Windows 2013, v22.0 (IBM Corp., Armonk, NY), and statistical significance used a two-tailed *p*-value < 0.05.

## Results

Demographic characteristics for the Continuing Promise 2011 ship’s population provided from personnel rosters, the anonymous weekly questionnaire, and the clinic case series are described in Table 1. The ship’s population was predominately male (70.7%), active duty Navy (76.6%), and enlisted rank (63.3%). Distributions were similar between personnel rosters and weekly surveillance questionnaires.

**Table 1:** Continuing Promise 2011 Population and Sample Demographics.

Characteristic	Ship Population	Self-reported TD Cases	Clinic-based TD Case Series
	(N = ~900, weekly avg)	(N = 3,156; avg 150 surveys/week)	(N = 193)
**Age, median (IQR)**	N/D	31 (24–36)]	32 (25–36)
**Gender, n (%)**			
Male	636 (70.7)	2,038 (64.6%)	129 (66.8%)
Female	264 (29.3%)	1,030 (32.6%)	63 (32.6%)
Missing	N/A	88 (2.8%)	1 (0.5%)
**Branch of Service, n (%)**			
Navy	689 (76.6%)	2,502 (79.3%)	N/A
Air Force	42 (4.7%)	194 (6.1%)	N/A
Army	11 (1.2%)	70 (2.2%)	N/A
NGO	54 (6.0%)	214 (6.8%)	N/A
Other	104 (11.6%)	124 (3.9%)	N/A
Missing	N/A	52 (1.6%)	N/A
**Crew Type, n (%)**			
Enlisted	569 (63.3%)	1,946 (61.7%)	93 (48.2%)
Officer	203 (22.5%)	671 (21.3%)	46 (23.8%)
Civilian (NGO/CIVMAR)	127 (14.1%)	269 (8.5%)	34 (17.6%)
**Missing, n (%)**	N/A	270 (8.6%)	20 (10.4%)

IQR—interquartile range; N/D—not described; N/A—not available; NGO—non-governmental organization; CIVMAR—civil marine service; TD—travelers’ diarrhea

**Fig 1:**
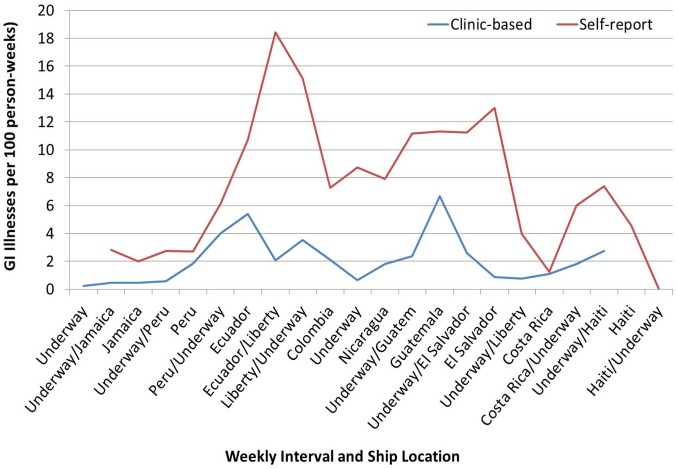
Epidemiologic Curve of Travelers’ Diarrhea Incidence by Week from Self-Reported Questionnaire (Active Surveillance) and Clinic Visits (Passive Surveillance).

Diarrheal disease incidence varied by method of data collection. Based on passive surveillance data collected from the clinic-based DNBI rates, acute GI illness was a leading reason for visiting the ship’s medical clinic, with an average incidence of 2.1 cases per 100 p-weeks, followed by dermatologic complaints (1. 9/100 p-weeks) and respiratory complaints (1.5/100 p-weeks) (*Riddle MS*, *manuscript in review*). Active surveillance via the weekly questionnaire resulted in collection of 3,156 questionnaires with an estimated average weekly incidence of 7.4 cases per 100 p-weeks. Incidence of acute GI illness appeared to vary by ship destination, with peaks associated on or after missions in Peru and Guatemala, as well as after liberty periods in Ecuador and Costa Rica (Fig 1).

Risk factor analysis based on data collected from the active weekly surveillance activity did not identify any demographic factors (e.g., gender, branch of service, and crew type) associated with increased rates of acute GI illness (Table 2). Eating off-ship in the prior week was significantly associated with GI illness (adjusted OR [aOR] 2.4, 95% confidence interval [CI] 1.8, 3.2). Among those that reported eating off-ship seafood consumption was weakly associated with increased risk (aOR 1.7, 95% CI 1.0, 2.8), whereas ice consumption off-ship had an inverse association with risk (aOR 0.3, 95% 0.1, 0.8) (Table 2). Due to lack of ability to assign country-specific exposure based on variation in incubation period as well as weekly survey and country mission timing misalignment, risk factor analysis was not stratified by country/high risk exposure period.

**Table 2:** Bivariate and Multivariate Analysis of Self-reported Travelers’ Diarrhea from Active Surveillance (Weekly Questionnaire, N = 3,156).

Characteristic/Risk Factor, n (%) unless otherwise specified	(+) GI Illness, N = 233 (7.4%)	(-) GI Illness, N = 2,923 (92.6%)	Crude Odds Ratio (95% CI)	p	Adjusted Odds Ratio (95% CI)	p
**Age (mean) ± SD**	30.1 ± 9	31.4 ± 10	1.3 (-0.1, 2.6)	0.06	1.0 (1.0, 1.0)	0.2
**Gender**						
Male	151 (65.4%)	1,887 (66.5%)	referent	0.7		
Female	80 (34.6%)	950 (33.5%)	1.0 (0.7, 1.3)			
**Branch of Service**						
Navy	189 (82.2%)	2,313 (80.5%)	referent	0.8		
Air Force	15 (6.5%)	179 (6.2%)	1.03 (0.6, 1.8)			
Army	3 (1.3%)	67 (2.3%)	0.55 (0.2, 1.8)			
NGO	13 (5.7%)	201 (7)	0.79 (0.4, 1.4)			
Other	10 (4.3%)	114 (4)	1.07 (0.6, 2.1)			
**Military rank**						
Enlisted	141 (67.1%)	1,805 (67.5%)	referent			
Officer	54 (25.7%)	617 (23.1%)	1.12 (0.8, 1.6)			
**Off-ship in last week**						
No	27 (11.6%)	380 (13)	referent			
Yes	206 (88.4%)	2, 543 (87)	0.88 (0.6, 1.3)	0.5		
**Consumed off-ship food in last week**						
No	122 (52.4%)	2,183 (74.7%)	referent			
Yes	111 (47.6%)	740 (25.3%)	2.68 (2.1, 3.5)	<0.0001	2.4 (1.8, 3.2)	<0.0001
**Off-ship food type**						
None listed	varies	varies	referent			
Beef	48 (58.5%)	309 (52)	1.3 (0.8, 2.1)	0.3	1.5 (0.9, 2.4)	0.1
Ceviche	2 (2.4%)	22 (3.7%)	0.7 (0.2, 2.8)	0.6	0.8 (0.2, 3.5)	0.7
Chicken	52 (63.4%)	408 (68.7%)	0.8 (0.5, 1.3)	0.3	0.7 (0.4, 1.2)	0.2
Empanada	3 (3.7%)	54 (9.1%)	0.4 (0.1, 1.2)	0.1	0.4 (0.1, 1.3)	0.1
Ice	8 (9.8%)	144 (24.2%)	0.3 (0.2, 0.7)	0.003	0.3 (0.1, 0.8)	0.01
Salad	10 (12.2%)	129 (21.7%)	0.50 (0.3, 1.0)	0.05	0.7 (0.3, 1.7)	0.5
Seafood	44 (53.7%)	257 (43.3%)	1.5 (1.0, 2.4)	0.1	1.7 (1.0, 2.8)	0.03

NGO—non-governmental organization, SD—standard deviation, GI—gastrointestinal; CI—confidence interval, T—temperature; HR—heart rate

Acute GI illness had substantial impact on individuals’ work ability, with 201 days of lost duty recorded (62% of all lost duty days), and was significantly associated with an increased odds of seeing a healthcare provider (OR 7.2, 95% CI 5.0, 10.2) as compared to those who did not report GI illness. In addition, relative to those reporting other DNBI events, individuals reporting GI illness were also significantly associated with an increased odds of reporting a negative impact on ability to carry out the mission (38.6% reported illness having a negative impact; OR 5.2, 95% CI 3.8, 7.2). Factors associated with increased odds of lost duty days or being confined to room included vomiting (*p* = 0.002), feeling lightheaded or weak (*p* = 0.005), and being a food handler (*p* = 0.017). Per public health policy, ill food handlers are excluded from work to minimize risk to others.

In total, 193 people with acute GI illness reported to the ship’s medical clinic and met the case definition for TD; a convenience sub-sample of 48 subjects provided a stool sample for culture-independent pathogen identification. An additional three stool samples from acute GI illness were also submitted, but not captured in the case-enrollment population. Similar demographic features and clinical symptoms existed between subsets that did and did not provide clinical samples with regard to subjective and objective complaints of lightheadedness, abdominal pain, decreased urination, past antibiotic use, reporting going off-ship in the past week, eating from an unapproved source, physical exam findings (mucous membranes, abdominal exam), and prescribed antibiotic treatment (Table 3). Noted differences between those who did and did not submit a stool sample included a greater percentage of those who submitted a sample presenting with both subjective complaints of fever (p<0.0001) and actual measured fever (p = 0.008). No food handlers provided a stool sample. An oversampling from Peru and under-sampling from Guatemala was also noted.

**Table 3:** Characteristics of the Travelers’ Diarrhea Cases Seen in the Medical Clinic Stratified by Stool Sample Collection.

Characteristic Present, n (%)	Stool Sample	Stool Sample	p-value	Total
	Yes, n = 48	No, n = 145		(N = 193)
**Clinical Symptoms**				
Lightheaded / weak	21 (43.8%)	57 (39.3%)	0.6	78 (40.4)
Abdominal pain	29 (60.4%)	98 (67.6%)	0.4	127 (65.8%)
Subjective fever/chills	9 (18.8%)	5 (3.4%)	<0.0001	14 (7.3%)
Hematemesis	0 (0.0%)	3 (2.1%)	0.6	3 (1.6)
Blood in stool	2 (4.2)	3 (2.1)	0.6	5 (2.6)
Decreased urination	11 (22.9)	34 (23.4)	0.9	45 (23.3)
Not able to keep fluids down	11 (22.9)	23 (15.9)	0.3	34 (17.6)
Physical exam findings*				
Tachycardia (HR >100)	8 (16.7)	16 (11.0)	0.3	24 (12.4)
Elevated temperature (T > 99.5 °F)	13 (27.1)	23 (15.9)	0.08	36 (18.7)
Fever (T >100.4°F)	6 (12.5)	3 (2.1)	0.008	9 (4.7)
Orthostatic hypotension	2 (4.2)	10 (6.9)	0.7	12 (6.2)
Mucous membranes dry/tacky	3 (6.3)	11 (7.6)	1	14 (7.3)
Tenderness on abdominal exam	20 (41.7)	61 (42.1)	0.9	81 (42)
**Risk Factors**				
Food handler	0 (0.0)	14 (9.7)	0.02	14 (7.3)
Off-ship in past week	44 (91.7)	132 (91.0)	1	176 (91.2)
Ate food from unapproved source	34 (70.8)	94 (64.8)	0.5	128 (66.3)
Other people in berthing ill	14 (29.2)	57 (39.3)	0.2	71 (36.8)
Antibiotics taken in past month	6 (12.5)	18 (12.4)	1	24 (12.4)
**Treatment Provided**				
Antibiotic & loperamide prescribed	43 (89.6)	116 (80.0)	0.1	159 (82.4)
Loperamide alone prescribed	1 (2.1)	14 (9.7)	0.1	15 (7.8)
Placed on bed-rest/confined to room	19 (39.6)	56 (38.6)	0.7	75 (38.9)
**Country visited in the prior week**			0.001	
Jamaica	3 (6.3)	1 (0.7)		4 (2.1)
Peru	17 (35.4)	16 (11.0)		33 (17.1)
Ecuador	8 (16.7)	32 (22.1)		40 (20.7)
Colombia	1 (2.1)	7 (4.8)		8 (4.1)
Nicaragua	5 (10.4)	20 (13.8)		25 (13.0)
Guatemala	9 (18.8)	53 (36.6)		62 (42.8)
El Salvador	1 (2.1)	9 (6.2)		10 (5.2)
Costa Rica	3 (6.3)	4 (2.8)		7 (3.6)
Haiti	1 (2.1)	3 (2.1)		4 (2.1)

*Percentages may not add up as denominator may change based on missing or not performed

The laboratory results for enteric pathogen identification are listed in Table 4 and are presented as either combined or stratified by testing method. Of those TD cases that visited the ship’s clinic and provided a stool sample, 48% were caused by a bacterial pathogen—predominately ETEC. Multiple pathogens were identified frequently at around 10% for each independent PCR method, and 24% when both PCR methods were combined. No pathogens were identified in approximately 40% of cases via individual PCR methods or in 29% of cases when methods were combined. The only enteric viral pathogen identified was norovirus (both genogroups), which was the sole pathogen in approximately 12% (6/51) of the samples. Other bacterial pathogens identified included *Shigella sonnei*, *Campylobacter* spp., non-typhoidal *Salmonella* spp., *C*. *difficile*, and other pathogenic *E*. *coli* strains (e.g., Shiga toxin-producing *E*. *coli*, *E*. *coli* O157, EPEC, and EAEC. ETEC plus ‘other pathogen‘ was the most common result, and among ETEC, ST-producing and CS6-expressing strains were most common (Table 5).

**Table 4:** Pathogen Etiology Identified By Two Culture-Independent Methods.

Etiology	Conventional PCR, n (%): N = 51	Luminex GPP, n (%): n = 50	Combined, n (%): N = 51
**Sole pathogen identified**			
ETEC	11 (22%)	14 (28%)	11 (22%)
Norovirus	6 (12%)	5 (10%)	6 (12%)
*Shigella* spp.	3 (6%)	1 (2%)	3 (6%)
*Campylobacter* spp.	3 (6%)	3 (6%)	3 (6%)
EPEC	1 (2%)	NT	0
EAEC	2 (4%)	NT	0
*Salmonella* spp. (non-typhoidal)	0	0	0
*Clostridium difficile*	NT	2 (4%)	1 (2%)
Pathogen—Negative	20 (39%)	21(42%)	15 (29%)
Pathogen—Mixed	5 (10%)	4 (8%)	12 (24%)
**Pathogen combinations**	ETEC, EAEC	ETEC, Salmonella	ETEC, EAEC
	ETEC, NoV	ETEC, *E*. *coli* O157 (n = 2)	ETEC, Shigella
	Shigella, EAEC	ETEC, STEC, NoV	ETEC, NoV (n = 2)
	ETEC, EPEC (n = 2)		ETEC, Salmonella
			ETEC, *E coli* O157 (n = 2)
			ETEC, EPEC (n = 2)
			ETEC, STEC, NoV
			Shigella, EAEC
			EPEC, *C*. *difficile*

ETEC—enterotoxigenic *E*. *coli*; EAEC—enteroaggregative *E*. *coli*, EPEC—enteropathogenic *E*. *coli*, NoV—norovirus, NT—not tested, PCR—polymerase chain reaction, STEC—shiga-toxin producing *E*. *coli*; GPP—Luminex xTAG^®^ Gastrointestinal Pathogen Panel

**Table 5:** Toxin and colonization factor distribution of Enterotoxigenic *E*. *coli* by PCR.

CF/Toxin, n (%)	LT	ST	LTST	CF total
**CS6, CS14**	0	5 (33)	0	5 (33)
**CS6 only**	0	1(7)	1 (7)	2 (13)
**CS21**	0	1(7)	0	1 (7)
**CS2,CS3,CS21**	0	0	1 (7)	1 (7)
**CS2, CS3**	1 (7)	0	0	1 (7)
**Negative**	3 (20)	2 (13)	0	5 (33)
**Toxin total**	4 (27)	9 (60)	2 (13)	15 (100)

LT- heat labile toxin, ST—heat stable toxin, CF—colonization factor, CS—coli surface (antigen), PCR—polymerase chain reaction

## Discussion

Continuing Promise is a biennial HA/DR training mission to the regions of Central and South America and the Caribbean with the goals of conducting training, fostering subject matter expert exchanges, and providing medical, dental, veterinary, and engineering assistance to the participating host nations. [15] To our knowledge, no studies have been published to date that use a combination of active and passive public health surveillance efforts to describe the epidemiology associated with acute GI illness (e.g., travelers’ diarrhea) on a ship-based HA/DR mission. Here, we utilized both active and passive public health surveillance methods to show that TD was the leading cause of illness during the mission—a finding that is consistent with prior studies showing TD as the most common ailment affecting military travelers. [2] Also, similar to other types of deployments and military missions, TD was found to be under-reported in this setting; where for every case seen at the medical clinic, there were approximately 3–4 other cases not seeking care [2]; this supports the observation of gaps in traditional, passive surveillance methods. Given the observed morbidity associated with TD and other disease syndromes, consideration of active disease surveillance methods on future deployments may be important to identify disease threats and support disease mitigation efforts.

In addition to a more complete assessment of disease burden, the active surveillance activity during Continuing Promise 2011 allowed for an assessment of risk factors that identified country- and liberty-associated increased rates, as well as individual self-reported exposure of eating unapproved food off—ship (Fig 1, Table 2). Anecdotally, additional value of this active surveillance was observed during the first noted peak of acute GI illness aboard ship, when concerns circulated among the crew that the *ship’s* food was the cause of the reported GI illnesses. However, the self-report data acquired in this study demonstrated that, in fact, the greatest risk for acquiring TD was eating *off-ship*. This information was deemed critical to the ship’s Command and was used to provide evidence-based force health protection briefings.

Similar to other TD risk factor studies, efforts to identify specific high-risk foods were unrevealing, although seafood did appear to be associated with increased risk on this mission. [20,21] Surprisingly, salad and ice trended toward a protective effect, which is inconsistent with prior studies. [17,22–24] The lack of consistency and identification of high-risk foods could be explained by recall bias, as well as the non-homogenous food type exposure/availability during the different country visits, which would have a tendency of biasing towards the null. Expansion and standardization of food categories and off-ship activities present on the survey form would increase the amount of epidemiological information provided and could greatly enhance the value of such a survey in future deployment.

Laboratory identification of enteric pathogens involved three assay methods, including conventional and real-time (RT-)PCR as well as the multi-pathogen panel-based Luminex xTAG^®^ GPP assay. Not surprisingly, acute GI illness among personnel disembarking to countries in this region were predominantly due to diarrheagenic *E*. *coli*, as well as norovirus. [2,25,26] Culture-independent methods are being used increasingly to understand the etiology of acute GI infections across a variety of populations and settings. [27–30] Culture-independent/molecular methods provide a more expedient and simplified means of identifying pathogens in clinical samples as compared to conventional diagnostic approaches that often include bacterial culture (requiring a variety of different culture media and growth conditions), microscopy with and without stains, immunofluorescence, and stool antigen tests for detection of protozoa. With the growing use and licensure of culture-independent methods, it is now possible to rapidly and simultaneously identify a multitude of bacterial, protozoan, and viral diarrheal pathogens from a single sample that can be analyzed either fresh or frozen. However, it is recognized that challenges in interpretation of culture-independent results exist. The uncertain correlation between presence of pathogen nucleic acids and disease attribution for both bacterial and viral etiologies makes determination of causation difficult. In the present study, we observed co-infection rates of approximately 10% for the conventional and real-time PCR methods versus Luminex platform, and 24% overall when these methods were combined. The application of quantitative PCR diagnostics in future studies, where both cases and controls are tested, may provide better insight into the specificity of infection without sacrificing sensitivity. [31] Regardless, it would appear that ETEC in combination with another enteric pathogen was most frequently identified, supporting ETEC as a predominant cause of TD in this region. [2]

This study has both strengths and limitations worth discussion. First, the Continuing Promise 2011 HA/DR mission represented a unique deployment environment in that the ship’s population had close interactions with local populations (e.g. they were treating sick individuals from the community); this exposed the crew to microbes that they would not normally encounter were they to remain segregated from those foreign populations. Importantly, to our knowledge, this is the first study to use both passive and active surveillance methods to characterize the incidence, etiologies, and risk factors associated with TD on a U.S. military ship-based HA/DR mission. Not only did this dual surveillance methodology allow for a direct comparison of the two methods in terms of acquired epidemiological information (e.g., disease burden and risk factors), it also increased the scope of surveillance, which ultimately uncovered a significant amount of TD that would have otherwise remained unreported. This is especially notable given the study population, which was comprised of a large number of health care workers who often either self-treat or are treated by colleagues and thus would likely not be represented in the (passive) DNBI reports.

Limitations of this study include the relatively low percentage (~15%) and convenience sample of survey respondents, which may have resulted in an under- or miss-representation of disease burden and risk factors. This active surveillance effort was for the primary purpose of providing additional information on the health of the shipboard population, which because of the nature of the composition being primarily licensed health care providers; we anticipated lower health-care seeking behavior (e.g. more self-treating). However, the extent of this public health activity was balanced by the limited personnel able to perform this work (extra duty), and practical and pragmatic considerations including the avoidance of an overly intrusive activity during a high operational tempo mission. The convenience sample methods were intended to survey a representative sample of the ship’s population in terms of demographic and occupational specialties (e.g. nurses, physicians, medics, admin, logistics). The collection of surveys at the time of mustering to go off ship each week would likely bias towards “healthy” workers being surveyed, though respondents were asked about illness presently and in the prior week. This may have biased estimates towards increased risk by enrichment for personnel at risk, as well as in the other direction by not capturing individuals who were presently ill at the time of survey. Future studies could focus on increasing active surveillance participation so that a significantly larger proportion of the ship’s crew is represented, however such efforts need to be balanced by the additional impact that such active surveillance might have both on Preventive Medicine and Public Health personnel and the ship’s crew. Similarly, as this was an augmented public health effort, lack of planned resources and personnel could only support a small proportion of stool sample collection and processing for people presenting to the clinic with TD. However, the samples collected represent a fair spectrum of disease and country distribution to the region. Finally, culture-independent methods of pathogen identification were employed for convenience and to enhanced detection of more fastidious pathogens that may otherwise be more difficult to store, grow, and detect. These methods at present cannot adequately discern disease etiology in cases of copathogen detection. Improvements in future study designs could incorporate inclusion of non-TD controls and more quantitative assessments of pathogen nucleic acid levels in order to better estimate the contribution of particular pathogens to clinical disease.

## Conclusions

The combination of active and passive public health surveillance data with downstream laboratory testing is certainly valuable in terms of establishing the epidemiology and etiology of TD incurred during military deployments. Furthermore, weekly active surveillance with the anonymous questionnaire provides for an adaptive and dynamic method in which to identify and inform evidence-based force health protection responses using a more real-time approach. However, the absence of shipboard diagnostics limits the potential application of information to help address specific gastrointestinal threats as they occur. A rapid, easy-to-use, molecular diagnostic platform that is forward-deployed to military units afloat and ashore could provide timely information important to public health (e.g., early identification of an index norovirus or *Shigella* spp. case that could easily spread). Subsequent, tailored/individual treatment (e.g., for cases of *Campylobacter* spp. or non-typhoidal *Salmonella* spp. requiring differential therapy) would likely outweigh the associated costs.

Given the high incidence of TD during military deployment and its ability to negatively impact the mission, it is important to continue public health surveillance activities during all military deployments, regardless of destination or scope. Not all TD can be avoided; however, more research is required to aid development of effective prevention/mitigation strategies. Incidence rates and pathogen identification data obtained from this study may be used to inform future evidence-based Force Health Protection efforts as well as the development of effective diagnostics, therapeutics, and prophylactics, such as vaccines—tools that will not only be beneficial to the U.S. military and its partners, but to other traveler populations in general.

## Supporting Information

S1 File Anonymous Weekly Disease and Non-Battle Injury Survey Form. (PDF)

S2 File Detailed Microbiology Materials and Methods. (PDF)
