# Supplementary material for: Incidence, Etiology and Risk Factors for Travelers’ Diarrhea during a Hospital Ship-Based Military Humanitarian Mission: Continuing Promise 2011
Source: PLoS One. 2016 May 12;11(5):e0154830. doi: 10.1371/journal.pone.0154830 (PMC4865142; doi:10.1371/journal.pone.0154830)
Supplement: S1 File — (PDF) [file pone.0154830.s001.pdf]

CONTINUING PROMISE 2011
ENHANCED DNBI QUESTIONNAIRE

SERVICE<br>☐Navy ☐Marine Corps ☐Air Force<br>☐Army ☐ Other_______________	AGE	GENDER<br>☐M ☐F	TODAY'S DATE<br>_ _/ _ _/ _ _<br>mm dd yy
UNIT / DEPARTMENT ☐AirDet ☐BoatDet ☐DAS ☐DESRON<br>☐ DFA ☐ DMS ☐ DNS ☐ DSS ☐ MESRON ☐NMCB28<br>☐S-1 ☐S-2 ☐S-3 ☐S-6 ☐PAO/Band ☐Translators ☐Other_________			COUNTRY ARRIVED ABOARD USNS COMFORT<br>________________________
RANK: ☐E1 – E3 ☐E4 – E6 ☐E7 – E9 ☐O1 – O3 ☐O4 – O6 ☐ Civilian			
>>>> NUMBER (#) OF DAYS OFF SHIP IN PAST WEEK |__| <<<<			

CURRENTLY or in the PAST WEEK, have you had: (*PLEASE CHECK ALL THAT APPLY*) YES √

**1. Any respiratory infection (e.g. ☐runny nose, ☐cough, ☐sore throat)?**	☐
**2. Any skin disease (*Please check*: ☐rash, ☐boils, ☐athlete's foot, ☐sunburn, ☐other ________)?**	☐
**3. Any diarrhea (≥ 3 loose/watery stools in 24 hour period)?**	☐
**4. Any blood in stools?**	☐
**5. Any vomiting?**	☐
**6. Any gynecological problems? IF MALE, *please skip***	☐
**7. Any heat injury (*Please check*: ☐dehydration, ☐heat exhaustion, ☐heat illness)?**	☐
**8. Any injury related to sports and recreation?**	☐
**9. Any injury related to motor vehicle accidents?**	☐
**10. Any injury related to work or training (NON-Combat)?**	☐
**11. Any injury due to other causes not described in questions 7 – 10?**	☐
**12. Any eye problems (*Please check*: ☐pink-eye, ☐corneal abrasion, ☐vision problems)?**	☐
**13. Any sexually transmitted infection (e.g. chlamydia, gonorrhea, etc.)?**	☐
**14. Any mental health problem (☐depression, ☐anxiety, ☐stress>>describe________________)?**	☐
**15. Any fever?**	☐
**16. Any other medical or surgical problem not described above? (describe__________________)**	☐
**17. Did you eat/drink any non-US food off ship (☐seafood, ☐chicken, ☐beef, ☐salad, ☐ice)?**	☐
**18. Any encounter with snakes or venomous insects (e.g. ☐snakes, ☐spiders, ☐scorpions)?**	☐
**19. Have you been bitten by any mosquitoes?**	☐
**20. *DID YOU GO TO SICKBAY* in the past 7 days for any above illness/injury?**	☐

21. Please mark best response indicating impact of disease/injury on your mission ability/performance:

☐No impact ☐Minor degree of impact ☐Moderate degree of impact ☐Severe degree of impact

>>>>>>>>>>>>> If ANY impact, describe____

T H A N K   Y O U !
