# Supplementary material for: Incidence, Etiology and Risk Factors for Travelers’ Diarrhea during a Hospital Ship-Based Military Humanitarian Mission: Continuing Promise 2011
Source: PLoS One. 2016 May 12;11(5):e0154830. doi: 10.1371/journal.pone.0154830 (PMC4865142; doi:10.1371/journal.pone.0154830)
Supplement: S2 File — (PDF) [file pone.0154830.s002.pdf]

Detailed Microbiology Materials and Methods

Pathogen identification

Whole Stool Collection and Nucleic Acid Purification

For each enrolled subject providing a stool specimen, between 1-5 milliliters (mL, liquid) or grams (solid) of stool were collected and aliquoted into a sterile, leak-proof, 20-mL vial for (RT)-PCR-based analysis of enteric pathogens. Vials were stored on-board at -70 °C and were shipped to NHRC on dry ice. Stool samples were diluted to a 20% solution with phosphate-buffered saline (lacking Ca^{2+}/Mg^{2+}, Lonza, Walkersville, MD) and were subjected to RNA or DNA extraction using the Qiagen QIAamp Viral RNA Mini Kit or the QIAamp Fast DNA Stool Mini Kit (Qiagen, Valencia, CA). Purified nucleic acids were eluted into nuclease-free centrifuge tubes and stored at -80 °C along with the clinical samples.

NoV real-time, multiplex RT-PCR

Total RNAs were subjected to a one-step, real-time, TaqMan®, RT-PCR assay to detect norovirus genogroup (G)I and GII as stipulated by the CDC CaliciNet program.[1] Total reaction volumes of 25-microliters (µL) contained the AgPath-ID™ One-Step RT-PCR Kit reagents per manufacturer's instructions (Life Technologies, Grand Island, NY) with final concentrations of additional reagents as follows: 0.4 micromolar (µM) Cog1 and Cog2 primers; 0.8 µM RNP primers; 0.2 µM probes (IDT, Coralville, IA); and 0.5-2.0 micrograms (µg) (or 2.5 microliter [µL]) RNA. Thermal cycling parameters were as follows: 1 cycle at 45 °C for 10 minutes (min); 1 cycle at 95 °C for 10 min; and 45 cycles at 95 °C for 15 sec and 60 °C for 1 min. All samples were run in duplicate. The CDC protocol was modified by the EDSP to include an endogenous internal amplification control (IAC) for each sample (human RNase P, see all primer sequences

and final concentrations in **Supplemental Table 1**). Positive (norovirus GI and GII RNAs) and negative (water) controls were included on every plate. All real-time PCR assays described herein were performed and analyzed using the Applied Biosystems 7500 Fast Dx Real-time PCR system and associated SDS v1.4 software (Thermo-Fisher, Waltham, MA).

<u>Enteric viruses conventional multiplex RT-PCR</u>

The EDSP's enteric viruses conventional, multiplex RT-PCR assay was based on that published by Khamrin P et al. but modified to include only astrovirus, groups A and C rotavirus, sapovirus, and adenovirus.[2] Additionally, an exogenous, spike-in GFP IAC was added to the PCR master mix. RT was performed in 20-µL volumes using the Qiagen OmniScript RT kit with final reagent concentrations as follows: 0.5 mM dNTPs; 0.6 µM adenovirus, astrovirus, groups A and C rotavirus, and sapovirus primers; 0.5 Ū/µL RNase Inhibitor; 0.2 Ū/µL RT enzyme; and 0.5-2.0 µg (9 µL) RNA. RT was carried out for 1 hour at 50 °C, followed by five min. at 95 °C and then immediately placed on ice or stored at -80 °C. DNA amplification was also performed in 20-µL volumes using the Qiagen HotStar Taq Plus Master Mix Kit with final reagent concentrations as follows: 0.6 µM adenovirus, astrovirus, groups A and C rotavirus, and sapovirus primers; 0.075 µM GFP primers; 0.025 pg of GFP DNA; and 0.5-2.0 µg (2 µL) cDNA. Thermal cycling was carried out as follows: 1 cycle at 95 °C for 5 min; 35 cycles at 94 °C for 1min, 50 °C for 1min, and 72 °C for 1 min; and 1 cycle at 72 °C for 10 min. Amplified DNAs were immediately loaded onto a 2% agarose gel or were stored at -80 °C.

<u>Pathogenic *E. coli* conventional multiplex PCR</u>

The EDSP's Pathogenic *E. coli* conventional, multiplex PCR assay was based on that published by Nguyen TV et al. but modified to include an endogenous IAC, the 16S ribosomal RNA gene, *rrsA*.[3] Total reaction volumes of 25-μL contained Promega GoTaq Flexi Kit reagents (Madison, WI) with final concentrations as follows: 2 millimolar (mM) $MgCl_2$; 0.1 mM dNTPs; 0.2 μM each primer with the exception of vt1 and rrsA primers (0.4 μM and 0.1 μM, respectively); 1.25 units (Ū) polymerase (Pol); and 0.5-2.0 μg, (~2.5 μL) sample DNA. PCR was carried out as follows: 1 cycle at 96 °C for 4 min; 30 cycles at 94 °C for 20 sec, 55 °C for 20 seconds (sec), and 72 °C for 10 sec; and 1 cycle at 72 °C for 7 min. Amplified DNAs were immediately loaded onto a 2% agarose gel and run using BioRad (Hercules, CA) horizontal gel electrophoresis cells and power boxes, or were stored at -80 °C. For all conventional PCR assays described herein, select positive controls (pathogen DNAs or plasmids [Genscript, Piscataway, NJ]) and a negative control (water) were included in each run. Nucleic acids were visualized using the Hoefer MacroVue UV-20 Transilluminator (Holliston, MA) and the BioRad Gel Doc™ XR+ system.

ETEC Toxins and Colonization Factors conventional, multiplex PCR

The EDSP's ETEC Toxins and Colonization Factors conventional, multiplex PCR assay was based on that published by Nada RA et al. but modified to include the endogenous *rrsA* IAC in every multiplex component (four separate PCRs per sample).[4] Total reaction volumes of 20-μL contained Promega GoTaq Flexi Kit reagents with final concentrations as follows: 2 mM $MgCl_2$; 0.1 mM dNTPs; 1.25 Ū Pol; 0.5-2.0 μg (3 μL) sample DNA; and various primer concentrations (**Supplemental Table 2**). PCR was carried out as follows: 1 cycle at 94 °C for 4 min; 35 cycles at 94 °C for 1 min, 55 °C for 30 sec, and 72 °C 1 min; and 1 cycle at 72 °C for 5

min. Amplified DNAs were immediately loaded onto a 1.5% agarose gel or were stored at -80 °C.

Salmonella/Shigella/Campylobacter real-time, multiplex PCR

The EDSP's *Salmonella/Shigella/Campylobacter* real-time, multiplex PCR assay was based on that published by Wiemer, D et al. but modified to exclude the *Yersinia* primer/probe set and include the exogenous, spike-in green fluorescent protein (GFP, IDT) IAC.[5] Total reaction volumes of 25-µL contained Qiagen QuantiTect Multiplex PCR Kit (with ROX dye) reagents, with final concentrations as follows: 0.4 µM each primer and 0.2 µM each probe with the exception of the GFP probe, which was 0.04 µM; 0.1 nanograms GFP DNA; and 0.5-2.0 µg (2.5 µL) sample DNA. The thermal cycling program was as follows: 1 cycle of 15 min at 95 °C; 45 cycles of 1 min at 94 °C, 30 sec at 50 °C, and 30 sec at 72 °C; and 1 cycle of 10 min at 72 °C.

Vibrio cholera conventional, multiplex PCR

The EDSP's *Vibrio cholera* conventional, multiplex PCR assay was based on that published by Mehrabadi JF et al. but modified to include the exogenous, spike-in GFP IAC.[6] Final reaction volumes of 25-µL contained Promega GoTaq Flexi Kit reagents with final concentrations as follows: 2 mM $MgCl_2$; 0.2 mM dNTPs; 0.5 µM each primer with the exception of ctxA primers (0.1 µM); 0.5 picograms (pg) GFP DNA; 0.5 Ū Pol; and 0.5-2.0 µg (3 µL) sample DNA. Thermal cycling was carried out as follows: 1 cycle at 94 °C for 4 min; 35 cycles at 94 °C for 1 min, 55°C for 30 sec, and 72 °C 1 min; and 1 cycle at 72 °C for 5 min. Amplified DNAs were immediately loaded onto a 1.5% agarose gel or were stored at -80 °C.

Luminex xTAG® GPP multiplex PCR

Sample Pretreatment and Controls. Sample pretreatment was performed per manufacturer's instructions prior to nucleic acid extraction. Briefly, ~100 – 150 mg of stool (or 100 µL of liquid stool samples) was added to a Bertin SK38 tube containing 1 mL of easyMag Lysis Buffer and 10 µL xTAG MS2*. xTAG MS2 was added to all sample pretreatment tubes, *EXCEPT to the negative control tube, as an extraction/internal control. 250 µL of each pretreated sample was processed for total nucleic acids. The remaining pretreated solution was stored at -80°C for a maximum of thirty days.

At least one negative extraction control was included per batch of processed specimens. For batches of 1 – 30 samples, one negative control was included. For larger batches, 31 – 60 samples and 61 – 92 samples, two or three negative control are recommended respectively. The negative control replaces the addition of sample with 100 µL of lysis buffer. One positive extraction control was included per batch of processed specimens. Positive controls include known strains (ZeptoMetrix) or positive clinical samples for the targeted viruses, bacteria or parasites. Bacteriophage MS2 is the internal control for the assay.

Nucleic acid extraction. Total nucleic acid was extracted with a QIAamp® MinElute™ Media Kit (Qiagen®), per manufacturer's instructions. The sample input volume from the pretreatment step was 250 µL and final elution volume was 120 µL

Multiplex Amplification. For each sample, 10 µL of extracted nucleic acid was amplified in a single multiplex RT-PCR/PCR reaction. An additional negative control was included at this step, per manufacturer's instructions. For this negative control, 10 µL of RNase-free water replaced the addition of sample to the reaction master mix.

A single multiplex reaction identifies all targets in the panel.

Bead Hybridization. Following the amplification step, 5 µL of the RT-PCR product was then added to the hybridization/detection reaction containing the universal tag and Streptavidin, R-Phycoerythrin conjugate. NOTE: The xTAG GPP Bead Mix tube was vortexed for 30 seconds x 2 (total of 60 s vortexing) at the highest speed to disperse the beads prior to use. The beads were not sonicated as indicated in the manufacturer's protocol.

Data Acquisition and Analysis. Following completion of bead hybridization, the Luminex MagPix system sorts and reads each sample, generating a signal for each bead population. The values are analyzed to determine the presence or absence of each microbial target and controls in each sample against an analyte-specific cut-off. The xTAG Data Analysis Software for the Gastrointestinal Pathogen Panel (TDAS GPP) analyzes the data to provide a report summarizing which pathogens are present.

Supplemental Table 1. Primer and Probe Sequences				
**Assay**	**Name**	**Target gene**	**Sequence (5' to 3')**	**Ref**
Norovirus	Cog1-F	GI, vp1	CGYTGGATGCGITTYCATGA	[1]
	Cog1-R	GI, vp1	CTTAGACGCCATCATCATTYA C	[1]
	Cog2-F	GII, vp1	CARGARBCNATGTTYAGRTGG ATGAG	[1]
	Cog2-R	GII, vp1	TCGACGCCATCTTCATTCACA	[1]
	RNP-F	rnp	AGATTTGGACCTGCGAGCG	[7]
	RNP-R	rnp	GAGCGGCTGTCTCCACAAGT	[7]
	Ring1C	GI, vp1	56-FAM-AGATYGCGITCICCTGTCCA –3BHQ_1	[1]
	Ring2	GII, vp1	Cy5–TGGGAGGGCGATCGCAATCT–3BHQ_2	[1]
	RNP-P	rnp	Cy3–TTCTGACCTGAAGGCTCT GCGCG–3IAbRQSp	[7]
Enteric Viruses	Astro-F	rdrp	GGACTGCAAAGCAGCTTCGTG	[2]
	Astro-R	rdrp	GTGAGCCACCAGCCATCCCT	[2]
	Adeno-F	hexA-F	TTCCCCATGGCICAYAACAC	[2]
	Adeno-R	hexA-F	CCCTGGTAKCCRATRTTGTA	[2]
	RotaA-F	vp7	AAAGGATGGCCAACAGGATCAGT	[2]
	RotaA-R	vp7	GTATARAAHACTTGCCACCAT	[2]
	RotaC-F	vp7	CAAATGATTCAGAATCTATTG	[2]
	RotaC-R	vp7	GTTTCTGTACTAGCTGGTGAA	[2]
	Sapo-F	vp1	CTCGCCACCTACRAWGCBTGGTT	[2]
	Sapo-R	vp1	CMWWCCCCTCCATYTCAAACAC	[2]
	GFP353-F	gfp	TGAAATTCATCTGCACCACT	NHRC
	GFP353-R	gfp	CAAGCAAAAGAATGGCATC	NHRC
Salmonella Shigella Campylo-bacter	Sal-F	ttrR	AATTAGCCATGTTGTAATCTC	[5]
	Sal-R	ttrR	ATTGTTGATTCAGGTACAAAC	[5]
	Sal-P	ttrR	56-JOEN-CAAGTTCAACGCGCAATTTA-BHQ_1	[5]
	Shig-F	ipaH	CAGAAGAGCAGAAGTATGAG	[5]
	Shig-R	ipaH	CAGTACCTCGTCAGTCAG	[5]
	Shig-P	ipaH	TexRd-ACAGGTGATGCGTGAGACTG-IAbRQSp	[5]
	Campy-F	gyrA	CTATAACAACTGCACCTACTAAT	[5]
	Campy-R	gyrA	ATGAAATTTTTGCCAGTGGTG	[5]

	Campy-P	gyrA	56-FAM-CTTAATAGCCGTCACCCCAC–BHQ_1	[5]
	GFP77-F	gfp	AGATGACGGGAACTACAAG	NHRC
	GFP 77-R	gfp	CCTTCAGCTCGATTCTATT	NHRC
	GFP 77-P	gfp	Cy5-CACCTTCGAACTTGACTTCAGCGC-IAbRQSp	NHRC
ETEC Toxins/CFs mPCR1	STh-F	estA2-4	AATTGCTACTATTCATGCTTTCAGGAC	[4]
	STh-R	estA2-4	TCT TTT TCA CCT TTC GCT CAG G	[4]
	STp-F	estA1	ATGAAAAAGCTAATGTTGGCA	[4]
	STp-R	estA1	TTAATAACATCCAGCACAGGCA	[4]
	LT-F	eltB1	CATAATGAGTACTTCGATAGAGGAAC	[4]
	LT-R	eltB1	GAAACCTGCTAATCTGTAACCATCC	[4]
	rrsA-F (16S rRNA)	rrsA	CTCTTGCCATCGGATGTGCCCA	NHRC
	rrsA-R	rrsA	CCAGTGTGGCTGGTCATCCTCTCA	NHRC
ETEC Toxins/CFs mPCR2	mPCRgp1F (CFA/I)	cfaB	TGAGTGCTTCWGCAGTAGAGA	[4]
	CFA1-R	cfaB	CAGCAAGTTTAACAATTACTTTTTAGT	[4]
	mPCRgp-F (CS4)	csaB	TGAGTGCTTCWGCAGTAGAGA	[4]
	CS4-R	csaB	AAGTCACATCTGCGGTTGATAGAG	[4]
	mPCRgp-F (CS14)	csuA1	TGAGTGCTTCWGCAGTAGAGA	[4]
	CS14-R	csuA1	TACTATTCGAAACACCTGCCG	[4]
	cssBCS6F (CS6)	cssB	GGA GTG GTA AAT GCA GGA AAC T	[4]
	cssbCS6R	cssB	GTA CCA GAC GAA TAT CCG CTA TTA	[4]
	rrsA-F	rrsA	CTCTTGCCATCGGATGTGCCCA	NHRC
	rrsA-R	rrsA	CCAGTGTGGCTGGTCATCCTCTCA	NHRC
ETEC Toxins/CFs mPCR3	CS3F1 (CS3)	cstA	GGTCTTTCACTGTCAGCTATGAGTT	[4]
	CS3R1	cstA	TAATGTTAAATTATCCTGAGGAGC	[4]

			C	
	CS5F1 (CS5) csfA	csfA	GCGTGACACGTCAGCTAATATAAAC	[4]
	CS5_7R	csfA	GGCATTCATATCAATAGAAATATGAGAC	[4]
	CS7F (CS7)	csvA	TGCTCCCGTTACTAAAAATACG	[4]
	CS5_7R	csvA	GGCATTCATATCAATAGAAATATGAGAC	[4]
	rrsA-F	rrsA	CTCTTGCCATCGGATGTGCCCA	NHRC
	rrsA-R	rrsA	CCAGTGTGGCTGGTCATCCTCTCA	NHRC
ETEC Toxins/CFs mPCR4	CS2F (CS2)	cotA	TCTGCTCGTATCAATACCCAAGTT	[4]
	CS2R	cotA	GTGCCAGCGAATGAAACCTCTAAA	[4]
	mPCRgp3F (CS17/19)	csbA/ csdA	ACTCTRTCGCATTAACCTATTCT	[4]
	CS17_19R	csbA/ csdA	GTCACTTTCATCGGAATTTGCGAG	[4]
	CS8_21F (CS21)	lngA	TATGAGCCTKCTGGAAGTYATCAT	[4]
	CS21R	lngA	GTTATTACGCACTTCGTCTGGT	[4]
	mPCRgp3F (CS1/PCF071)	csoA	ACTCTRTCGCATTAACCTATTCT	[4]
	CS1_PCFO71R	csoA	CCCTGATATTGACCAGCTGTTAGT	[4]
	rrsA-F	rrsA	CTCTTGCCATCGGATGTGCCCA	NHRC
	rrsA-R	rrsA	CCAGTGTGGCTGGTCATCCTCTCA	NHRC
E. coli	EAEC-F	pCVD	CTGGCGAAAGACTGTATCAT	[3]
	EAEC-R	pCVD	CAATGTATAGAAATCCGCTGTT	[3]
	EHEC-F	vt1	GAAGAGTCCGTGGGATTACCG	[3]
	EHEC-R	vt1	AGCGATGCAGCTATTAATAA	[3]
	EHEC-F	vt2	ACCGTTTTTCAGATTTTRCACATA	[3]
	EHEC-R	vt2	TACACAGGAGCAGTTTCAGACAGT	[3]
	EHEC-F	eaeA	CACACGAATAAACTGACTAAAATG	[3]
	EHEC-R	eaeA	AAAAACGCTGACCCGCACCTAAAT	[3]
	EIEC-F	ial*	CTGGTAGGTATGGTGAGG	[3]
	EIEC-R	ial*	CCAGGCCAACAATTATTTCC	[3]
	EPEC-F	bfpA	TTCTTGGTGCTTGCGTGTCTTTT	[3]
	EPEC-R	bfpA	TTTTGTTTGTTGTATCTTTGTAA	[3]
	ETEC-F	eltB	TCTCTATGTGCATACGGAGC	[3]

	ETEC-R	eltB	CCATACTGATTGCCGCAAT	[3]
	ETEC-F	estA	GCTAAACCAGTARGGTCTTCAAAA	[3]
	ETEC-R	estA	CCCGGTACARGCAGGATTACAACA	[3]
	GFP77-F	gfp	AGATGACGGGAACTACAAG	NHRC
	GFP 77-R	gfp	CCTTCAGCTCGATTCTATT	NHRC
V. cholera	ctxA-F	ctxA	GGTCTTATGCCAGAGGACAG	[6]
	ctxA-R	ctxA	GTTGGGTGCAGTGGCTATAAC	[6]
	tcpA-F	tcpA	ATTCTTGGTGATCTCATGATAAGG	[6]
	tcpA-R	tcpA	TTAATTCACCACAAATATCTGCC	[6]
	ompW-F	ompW	CACCAAGAAGGTGACTTTATTGTG	[6]
	ompW-R	ompW	GAACTTATAACCACCCGCG	[6]
	GFP77-F	gfp	AGATGACGGGAACTACAAG	NHRC
	GFP 77-R	gfp	CCTTCAGCTCGATTCTATT	NHRC

NHRC, Naval Health Research Center

Supplemental Table 2a: ETEC Toxins Master Mix

Component	µL/Rxn	Final	Amplicon size
Nuclease-free H_2O	4.4	--	
Promega GoTaq Flexi 5x PCR	5	1 X	
$MgCl_2$ (25 mM)	2	2 mM	
dNTP (10 mM)	1	100µM/ea	
10 µM rrsA-F (16S rRNA)	0.18	1.8 pmol	105 bp
10 µM rrsA-R	0.18	1.8 pmol	
30 µM estA2-4F (SThF)	0.5	15 pmol	133 bp
30 µM estA2-4R (SThR)	0.5	15 pmol	
30 µM estA1-F (STpF)	1	30 pmol	239 bp
30 µM estA1-R (STpR)	1	30 pmol	
30 µM eltB1-F (LTF)	2	60 pmol	402 bp
30 µM eltB1-R (LTR)	2	60 pmol	
Promega GoTaq Flexi DNA	0.25	1.25 U	
**TOTAL VOLUME**	**20**		

Supplemental Table 2b: ETEC Colonization Factors Master Mix 1

Component	µL/Rxn	Final	Amplicon size
Nuclease-free H_2O	3.4	--	
Promega GoTaq Flexi 5x PCR	5	1 X	
$MgCl_2$ (25 mM)	2	2 mM	
dNTP (10 mM)	1	100µM/ea	
10 µM rrsA-F (16S rRNA)	0.18	1.8 pmol	105 bp
10 µM rrsA-R	0.18	1.8 pmol	

30 µM mPCRgp1F (CFA/I)	1	30 pmol	204
30 µM CFA1-R	1	30 pmol	
30 µM mPCRgp1F (CS4)	1	30 pmol	300
30 µM CS4-R	1	30 pmol	
30 µM mPCRgp1F (CS14)	1	30 pmol	357
30 µM CS14-R	1	30 pmol	
30 µM cssBCS6F (CS6)	1	30 pmol	416
30 µM cssbCS6R	1	30 pmol	
Promega GoTaq Flexi DNA	0.25	1.25 U	
**TOTAL VOLUME**	**20**		

Supplemental Table 2c: ETEC Colonization Factors Master Mix 2

Component	µL/Rxn	Final	Amplicon size
Nuclease-free H_2O	5.4	--	
Promega GoTaq Flexi 5x PCR	5	1 X	
$MgCl_2$ (25 mM)	2	2 mM	
dNTP (10 mM)	1	100µM/ea	
10 µM rrsA-F (16S rRNA)	0.18	1.8 pmol	105 bp
10 µM rrsA-R	0.18	1.8 pmol	
30 µM CS3F1 (CS3)	1	30 pmol	136 bp
30 µM CS3R1	1	30 pmol	
30 µM CS5F1 (CS5)	1	30 pmol	235 bp
30 µM CS5 7R	1	30 pmol	
30 µM CS7F (CS7)	1	30 pmol	418 bp
30 µM CS5 7R	1	30 pmol	
Promega GoTaq Flexi DNA	0.25	1.25 U	
**TOTAL VOLUME**	**20**		

Supplemental Table 2d: ETEC Colonization Factors Master Mix 3

Component	µL/Rxn	Final	Amplicon size
Nuclease-free H_2O	3.4	--	
Promega GoTaq Flexi 5x PCR	5	1 X	
$MgCl_2$ (25 mM)	2	2 mM	
dNTP (10 mM)	1	100µM/ea	
10 µM rrsA-F (16S rRNA)	0.18	1.8 pmol	105 bp
10 µM rrsA-R	0.18	1.8 pmol	
30 µM CS2F (CS2)	1	30 pmol	140 bp
30 µM CS2R	1	30 pmol	
30 µM mPCRgp3F (CS17/19)	1	30 pmol	169 bp
30 µM CS17 19R	1	30 pmol	
30 µM CS8 21F (CS21)	1	30 pmol	292 bp
30 µM CS21R	1	30 pmol	

30 µM mPCRgp3F	1	30 pmol	334 bp
30 µM CS1_PCFO71R	1	30 pmol	
Promega GoTaq Flexi DNA	0.25	1.25 U	
**TOTAL VOLUME**	**20**		

REFERENCES

1. Vega E, Barclay L, Gregoricus N, Williams K, Lee D, Vinje J, 2011. Novel surveillance network for norovirus gastroenteritis outbreaks, United States. Emerg Infect Dis 17: 1389-95.
2. Khamrin P, Okame M, Thongprachum A, Nantachit N, Nishimura S, Okitsu S, Maneekarn N, Ushijima H, 2011. A single-tube multiplex PCR for rapid detection in feces of 10 viruses causing diarrhea. J Virol Methods 173: 390-3.
3. Nguyen TV, Le Van P, Le Huy C, Gia KN, Weintraub A, 2005. Detection and characterization of diarrheagenic Escherichia coli from young children in Hanoi, Vietnam. J Clin Microbiol 43: 755-60.
4. Nada RA, Shaheen HI, Touni I, Fahmy D, Armstrong AW, Weiner M, Klena JD, 2010. Design and validation of a multiplex polymerase chain reaction for the identification of enterotoxigenic Escherichia coli and associated colonization factor antigens. Diagn Microbiol Infect Dis 67: 134-42.
5. Wiemer D, Loderstaedt U, von Wulffen H, Priesnitz S, Fischer M, Tannich E, Hagen RM, 2011. Real-time multiplex PCR for simultaneous detection of Campylobacter jejuni, Salmonella, Shigella and Yersinia species in fecal samples. Int J Med Microbiol 301: 577-84.
6. Mehrabadi JF, Morsali P, Nejad HR, Imani Fooladi AA, 2012. Detection of toxigenic Vibrio cholerae with new multiplex PCR. J Infect Public Health 5: 263-7.
7. Boddicker JD, Rota PA, Kreman T, Wangeman A, Lowe L, Hummel KB, Thompson R, Bellini WJ, Pentella M, Desjardin LE, 2007. Real-time reverse transcription-PCR assay for detection of mumps virus RNA in clinical specimens. J Clin Microbiol 45: 2902-8.
